# Early preterm delivery due to placenta previa is an independent risk factor for a subsequent spontaneous preterm birth

**DOI:** 10.1186/1471-2393-12-82

**Published:** 2012-08-10

**Authors:** Offer Erez, Lena Novack, Vered Klaitman, Idit Erez-Weiss, Ruthy Beer-Weisel, Doron Dukler, Moshe Mazor

**Affiliations:** 1 Department of Obstetrics and Gynecology, Soroka University Medical Center, School of Medicine, Faculty of Health Sciences, Ben Gurion University of the Negev, P O Box 151, Beer Sheva, 84101, Israel; 2 Departments of Epidemiology, Faculty of Health Sciences, Ben Gurion University of the Negev, Beer Sheva, Israel; 3 Departments of Family Medicine, Faculty of Health Sciences, Ben Gurion University of the Negev, Beer Sheva, Israel

**Keywords:** Preterm birth, Placenta, Recurrent preterm delivery, Vaginal bleeding, Short cervix, Placenta previa

## Abstract

**Background:**

To determine whether patients with placenta previa who delivered preterm have an increased risk for recurrent spontaneous preterm birth.

**Methods:**

This retrospective population based cohort study included patients who delivered after a primary cesarean section (n = 9983). The rate of placenta previa, its recurrence, and the risk for recurrent preterm birth were determined.

**Results:**

Patients who had a placenta previa at the primary CS pregnancy had an increased risk for its recurrence [crude OR of 2.65 (95% CI 1.3-5.5)]. The rate of preterm birth in patients with placenta previa in the primary CS pregnancy was 55.9%; and these patients had a higher rate of recurrent preterm delivery than the rest of the study population (p < .001). Among patients with placenta previa in the primary CS pregnancy, those who delivered preterm had a higher rate of recurrent spontaneous preterm birth regardless of the location of their placenta in the subsequent delivery [OR 3.09 (95% CI 2.1-4.6)]. In comparison to all patients with who had a primary cesarean section, patients who had placenta previa and delivered preterm had an independent increased risk for recurrent preterm birth [OR of 3.6 (95% CI 1.5-8.5)].

**Conclusions:**

Women with placenta previa, who deliver preterm, especially before 34 weeks of gestation, are at increased risk for recurrent spontaneous preterm birth regardless to the site of placental implantation in the subsequent pregnancy. Thus, strict follow up by high risk pregnancies specialist is recommended.

## Background

Placenta previa is a risk factor for preterm birth, and contributes to about 5% of all preterm deliveries. [1] The prevalence of placenta previa is 0.3-0.5% of pregnancies [2-10], and the risk for this complication increases according to the number of prior cesarean deliveries [11-14]. Placenta previa is associated with an increased maternal morbidity including the need for blood and blood products transfusion, urgent cesarean section, and cesarean hysterectomy. Moreover, a higher rate of perinatal mortality and morbidity, especially respiratory distress syndrome and anemia are associated with this abnormal placentation [15,16].

Most of the patients with placenta previa are delivered preterm [4,17], and these deliveries are regarded as indicated preterm births due to excessive maternal hemorrhage. Nevertheless, recent evidence suggests that other mechanisms aside bleeding may lead to preterm birth in women with placenta previa [18,19]. Patients with placenta previa who delivered preterm had a higher rate of intra-amniotic infection/inflammation than those who delivered at term [18], suggesting that similarly to spontaneous preterm birth, intra-amniotic infection or inflammation may contribute to the process of preterm parturition in patients with placenta previa. Moreover, women with this complication who had a short cervical length have an increased risk to deliver preterm [20-22]. Thus, the mechanisms leading to spontaneous preterm parturition may play a similar role in patients with placenta previa who deliver prematurely.

Placenta previa is a recurrent pregnancy complication; reports suggest a recurrence rate of 2.3-3.2% [23,24]. The underlying mechanisms leading to this are not completely understood. Yet, it is not clear from the literature whether patients with placenta previa who deliver preterm are at increased risk for recurrent preterm birth. The objective of this study was to determine whether women with placenta previa who delivered preterm are at increased risk for recurrent preterm birth in the subsequent pregnancy.

## Methods

### Study population, selection of patients

This is a retrospective population based cohort study including all women who delivered subsequent to a primary cesarean section (CS) during the study period (1988–2010) at the “Soroka University Medical Center”, a regional tertiary medical center where all the births take place, and met the inclusion criteria. This cohort (n = 9983) was divided into two groups according to the site of placentation at the primary CS: Patients with placenta previa comprised the study group (n = 297), and those with normal placental insertion served as the comparison group (n = 9686).

The patients were identified in a computerized database including all data concerning demographic characteristics, medical and obstetric history, pregnancy outcomes as well as, maternal and neonatal morbidity and mortality of all the deliveries at our medical center.

Women who lacked minimal prenatal care (less than three visits in prenatal clinic), those with multiple gestations, and parturient carrying a fetus with known chromosomal or anatomical anomalies were excluded from the study. The Institutional Review Board of Soroka University Medical Center approved the study.

### Outcome variables and clinical definitions

Parity groups were defined in the following order: primipara, multipara (2–5 deliveries) and grand multipara (6 or more deliveries). Gestational age was determined by date of last menstrual period when reliable and sonographic confirmation carried out by the first 20 weeks of gestation and/or first trimester sonographic measurement of crown- rump length. Hypertensive disorders of pregnancy were defined according the American College of Obstetrics and Gynecology (ACOG) criteria [25]. Placenta previa was defined as a placenta that partially or fully covers the internal cervical os, or when the lower placental edge lies within 20 mm from it [3]. The location of the placenta was diagnosed prenatally by ultrasound examination and verified during the cesarean delivery. Preterm delivery was defined as delivery before complete 37 weeks of gestation, early preterm birth was defined as delivery <34 weeks of gestation, and late preterm birth was defined as delivery between 34 to 36.9 weeks of gestation. Small for gestational age (SGA) was defined as birthweight below the 10^th^ percentile [26].

### Statistical analysis

The rate of placenta previa, its recurrence, and the risk for preterm delivery at the subsequent delivery following a placenta previa at the primary CS pregnancy, were determined as primary outcomes.

Maternal demographic characteristics, peripartum complications and perinatal outcome were compared between women with and without placenta previa. Parametric and non-parametric statistics were used for continues variables according to their distribution. Chi-square and Fisher exact test were used to compare categorical variables. Variables found to be significantly associated with placenta previa and preterm birth in the univariate analysis were included in a multiple logistic regression. A two tailed P value of 0.05 was considered significant. Analysis was done by SPSS package (Chicago, IL, USA) and SAS software version 9.2 (Cary, NC, USA).

## Results

The rate of placenta previa in the primary CS pregnancy was 3.0% (297/9983), and 1.08% (108/9983) in the subsequent pregnancy. The recurrence rate of placenta previa among patients who had this complication at the primary CS pregnancy was higher than among those without it [placenta previa- 2.69% (8/297) vs. normal placentation- 1.03% (100/9686) crude odds ratio (OR) of 2.65 (95% CI 1.2-5.7)].

Women with placenta previa had a higher mean maternal age and grand multiparity rate than those with normally implanted placentae in both pregnancies. The rate of prior preterm birth did not differ between patients with placenta previa and those with normal placentation in neither of the pregnancies (Table 1).

**Table 1:** Demographics and clinical characteristics of the study groups in both pregnancies

**Measure**	**Index Pregnancy**			**2**^**nd**^**pregnancy**		
	**Normal placentation**	**Placenta Previa**	**P-value**	**Normal placentation**	**Placenta Previa**	**P-value**
	**N = 9686**	**N = 297**		**N = 9875**	**N = 108**	
Maternal Age (years)	27.18 ± 5.20	29.02 ± 5.04	<.001	30.03 ± 5.59	33.06 ± 4.92	<.001
Gravidity						
1	42.2 (4086)	13.1 (39)	<.001	0.0 (0)	0.0 (0)	<.001
2-5	42.3 (4096)	60.6 (180)		77.0 (7603)	60.2 (65)	
6+	15.5 (1504)	26.3 (78)		22.9 (2263)	39.8 (43)	
Parity						
1	53.5 (4995)	20.4 (58)	<.001	0.0 (0)	0.0 (0)	<.001
2-5	38.0 (3552)	65.8 (187)		87.1 (8189)	79.2 (80)	
6+	8.5 (791)	13.7 (39)		12.4 (1169)	20.8 (21)	
Prior preterm birth	17.0 (602/3541)	14.1 (26/185)	.297	21.7 (2144/9875)	22.1 (24/108)	.898
Infertility treatments	5.5 (531)	8.1 (24)	.07	7.2 (709)	12.0 (13)	.06
Mild preeclampsia	5.4 (520)	1.0 (3)	<.001	3.5 (345)	2.8 (3)	1.0
Severe preeclampsia	5.3 (512)	1.7 (5)	.003	1.7 (164)	0.9 (1)	1.0
Chronic Hypertension	2.5 (242)	1.3 (4)	.25	3.0 (295)	2.8 (3)	1.0
GDM class A	6.4 (619)	7.7(23)	.346	6.6 (651)	5.6 (6)	.85
GDM class B-R	2.1 (201)	2.7 (8)	.41	2.7 (265)	1.9 (2)	1.0
Hydramnios	6.5 (632)	5.4 (16)	.55	5.5 (540)	4.6 (5)	1.0
Oligohydramnios	6.5 (627)	2.7 (8)	.005	2.7 (265)	1.9 (2)	1.0
Preterm Delivery	16.6 (1611)	55.9 (166)	<.001	9.6 (947)	51.9 (56)	<.001
Cesarean section	100.0 (9686)	100 (297)	---	52.2 (5151)	96.3 (104)	<.001

Data presented as percent (number) or mean ± standard deviation.

GDM- gestational diabetes.

**Figure 1:**
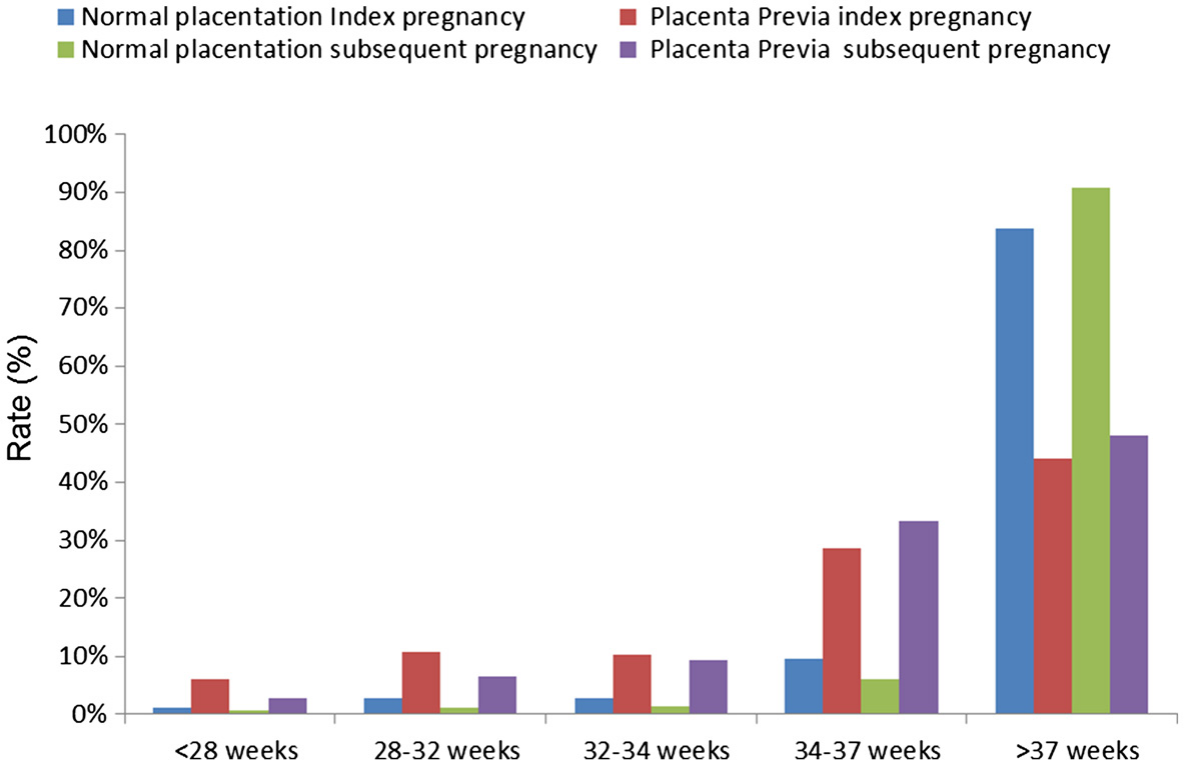
Distribution of gestational age at delivery among the study groups in each pregnancy.

The rate of preterm delivery among patients with placenta previa in the primary CS pregnancy was 55.9% (166/297) and 51.9% (56/108) among those who had placenta previa in the subsequent birth. In both pregnancies studied, the rate of severe prematurity (<32 weeks of gestation) was higher among women with placenta previa than in those with normal placentation [primary CS - normal placentation 4.1% (395/9686) vs. placenta previa 16.9% (50/297), OR 4.13 (95%CI 3.15-5.41), p < .001; subsequent delivery- normal placentation 2% (195/9875) vs. placenta previa 9.3% (10/108), OR 5.07 (95%CI 2.45-10.18), p < 0.001] (Figure 1).

**Figure 2:**
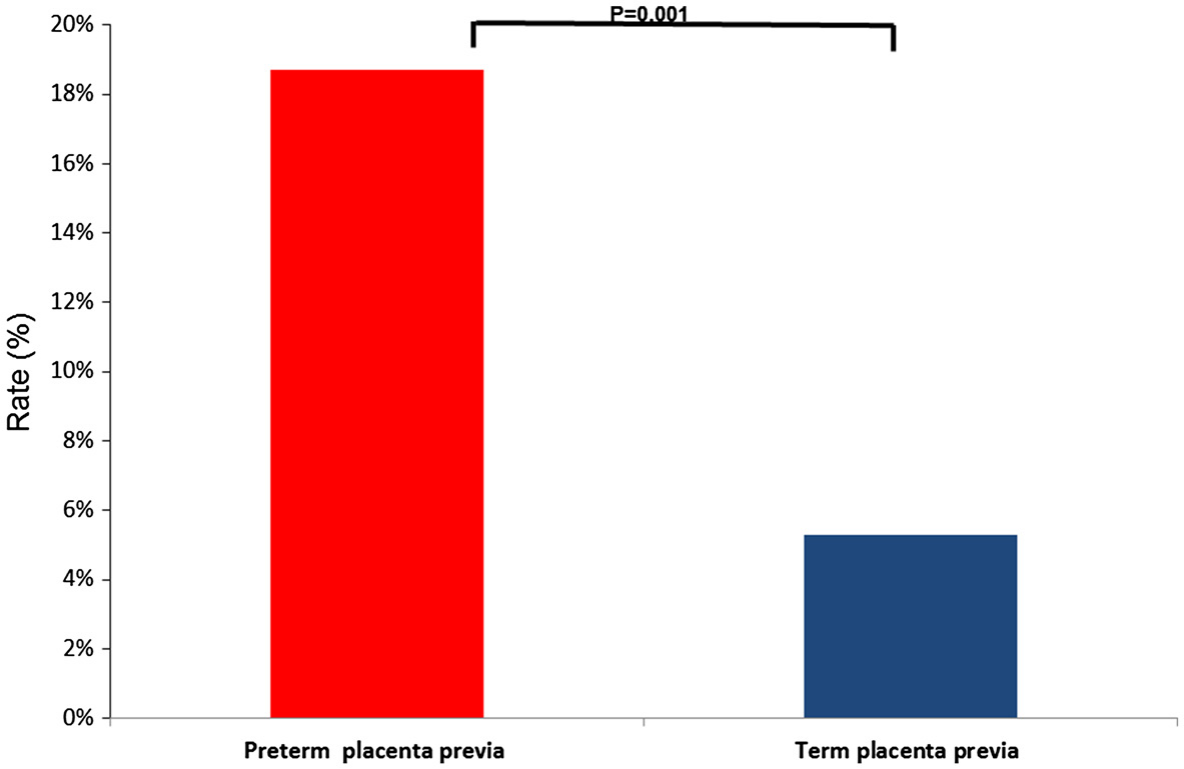
Recurrence of preterm delivery among patients with placenta previa who delivered preterm or at term in the primary CS pregnancy.

Women with placenta previa who delivered preterm at the primary CS pregnancy had a higher rate of recurrent preterm birth in the following delivery than the rest of the study population [preterm placenta previa: 18.7% (31/166) vs. study cohort: 9.9% (972/9817), p < .001, OR = 2.09 (95%CI 1.41-3.11)]. Moreover, among patients with a placenta previa in the primary CS pregnancy, those who delivered preterm had an OR = 3.09 (95% CI 2.1-4.6) for recurrent preterm birth in the subsequent pregnancy in comparison to those who delivered at term (Figure 2). Women with placenta previa who delivered before 34 weeks of gestation in the primary CS pregnancy had a higher risk to deliver preterm in comparison to those with placenta previa who delivered at term [early preterm delivery 25% (20/80) vs. term delivery 5.3% (7/131), p < .001, RR 4.7, 95% CI 2.07-10.57). However, women with placenta previa who had a late preterm birth, had an increased but not statistically significant rate of recurrent preterm birth in comparison to those who delivered at term (late preterm birth 12.8% (11/86), p = .09).

In a multiple logistic regression model aimed to study the recurrence of preterm delivery in patients with placenta previa, regardless to site of placentation, preterm birth in the second pregnancy was introduced as an outcome. Previous preterm delivery due to preeclampsia, intrauterine growth restriction (IUGR), placenta previa, preterm prelabor rupture of membranes (PROM) and spontaneous preterm parturition were included as covariates. Placenta previa at the primary CS pregnancy on its own was not a risk factor for preterm birth at the subsequent pregnancy. However, Patients with placenta previa who delivered preterm in the primary CS pregnancy had an independent increased risk for spontaneous preterm birth in the subsequent delivery unrelated to the presence of placenta previa in that pregnancy (OR 3.60, 95% CI 1.52-8.51) (Table 2).

**Table 2:** Risk factors at the index pregnancy for recurrent preterm birth at the subsequent delivery

** Factor**	**Odds Ratio ( 95% confidence interval)**
Spontaneous preterm birth	4.38 (3.71-5.17)
Preterm placenta previa	3.60 (1.52-8.51)
Preterm severe preeclampsia	2.82 (1.66-4.78)
Preterm IUGR	1.48 (1.003-2.18)
Severe preeclampsia	1.56 (1.01-2.53)
IUGR	1.52 (1.18-1.96)
Placenta previa	0.71 (.33-1.54)
Maternal age	1.03 (1.01-1.04)

## Discussion

*Principal findings of this study*: Placenta previa is a recurrent pathology. Preterm birth in patients with placenta previa is an independent risk factor for a recurrent spontaneous preterm delivery in the subsequent pregnancy regardless to the site of placental implantation.

Placenta previa is a risk factor for preterm birth [15,17]. Indeed, about 60% of the patients with placenta previa in our study delivered preterm, mainly due to vaginal bleeding. It has been proposed that in cases of placenta previa a certain degree of spontaneous placental separation is an inevitable consequence of the formation of the lower uterine segment and cervical dilatation, leading to severe hemorrhage [27] and indicated preterm birth. Moreover, there is evidence to support the delivery of women with placenta previa between 36 to 37 weeks, this practice is based upon the findings of Ananth et al. [16], who demonstrated that women with placenta previa have an increased perinatal mortality after 37 weeks of gestation. Collectively, the combination of severe vaginal bleeding that endangers the mother and the increased unexplained stillbirth in these patients after 37 weeks contributes to the high proportion of preterm deliveries reported in patients with placenta previa. Nevertheless, it is not clear from the current literature whether the increased risk for preterm birth is limited to the pregnancy affected by placenta previa or does it affect the subsequent ones as well.

In light of our findings and those of others [24], we raise the question of whether preterm delivery due to placenta previa is really an indicated preterm birth. Could it be that the premature bleeding in patients with placenta previa is the clinical presentation of a spontaneous preterm parturition? The answer to this question may be deduced from epidemiological and clinical studies regarding the association of placenta previa and the preterm parturition syndrome.

Shortening of the uterine cervix during gestation is a risk factor for preterm birth in patients with normal placentation [28,29], recent studies suggest that this is the same in patients with placenta previa [20-22]. Indeed among women with placenta previa, those who had a cervical length <30 mm at the third trimester had a higher rate of preterm delivery and a higher proportion of them required delivery due to hemorrhage in comparison to those with longer cervical length [21]. In addition, Ghi et al. [20] reported that patients with placenta previa who had emergency cesarean section due to bleeding at < 34 weeks of gestation had a significantly shorter cervical length than those who had elective cesarean delivery later during gestation. The authors concluded that a short cervix in patients with placenta previa may herald premature onset of labor and possible detachment of the placenta from its low insertion [20].

Vaginal bleeding can be the only manifestation of intra-amniotic infection and/or inflammation [30]. Indeed, among patients with placenta previa and vaginal bleeding the rate of intra amniotic infection was 5.7% and intra-amniotic inflammation was detected in 17.9% of these patients [19]. Moreover, among patients with placenta previa, those who had intra-amniotic infection or inflammation had a higher rate of delivery within 48 hours from admission and a lower mean gestational age at delivery than those without it [19]. In a different study, women with placenta previa who were admitted with an episode of preterm labor with intact membranes had a rate of 4.9% of intra-amniotic infection and 16.7% of intra-amniotic inflammation [18]. In addition, women with placenta previa who present with preterm labor and have intra-amniotic inflammation had a higher risk of intra-amniotic infection and a shorter admission to delivery interval. Thus, similarly to women with normal placentation, infection and or inflammation may be part of the mechanisms that prematurely activate the common pathway of parturition in patients with placenta previa, leading to preterm labor that is associated in some of the cases with vaginal bleeding and eventually progress to preterm birth.

Our finding that women with placenta previa who delivered preterm are at increased risk for spontaneous preterm birth in the subsequent delivery regardless to the site of placental implantation is novel. Preterm delivery is a recurrent disease; both spontaneous and indicated preterm births are associated with an increased risk for recurrence in subsequent pregnancies [31,32]. Moreover, there is an inverse correlation between the gestational age at delivery and the risk for recurrent preterm birth [31,32] and patients who experienced a spontaneous preterm parturition, have a higher recurrence rate than the general population for any gestational age in which the preterm delivery occurred.

Among multiparous patients, a prior preterm birth is the most prominent risk factor for a recurrent preterm delivery [33,34]. This is important since in our cohort the rate of previous preterm birth did not differ significantly between the study groups; suggesting that the independent risk for recurrent preterm birth in women with placenta previa who delivered preterm is a novel observation that does not the result from their obstetric history. Similarly to spontaneous preterm birth, placenta previa is a recurrent pathology. In our cohort, the recurrence rate of placenta previa was 2.7%. This is in accord with previous reports that the recurrence rate of placenta previa in different population varies from 2.3% to 3.2% [23,24,35]. The novel finding of this study that women who had a preterm delivery as a result of placenta previa have an independent increased risk (OR 3.6) for a spontaneous preterm birth in the subsequent pregnancy, even in the absence of recurrent placenta previa, is of importance. This odds ratio is higher than that for repeated placenta previa (2.65), and is similar to the risk of recurrent preterm birth in patients who had a previous spontaneous preterm birth [36]; further supporting the assumption that the mechanisms leading to spontaneous preterm birth may be also involved in preterm parturition among patients with placenta previa.

The shortcoming of this study is its retrospective nature, and the inherited weakness of studies based on a large dataset. In addition due to the structure of the database and the long time period the data was collected information regarding cervical length in our patients is missing. However, its population based scope and the large number of cases allow us to identify valuable information regarding the epidemiology of preterm birth among patients with placenta previa.

## Conclusions

We present herein evidence that preterm delivery in patients with placenta previa, especially if it occurs before 34 weeks of gestation, is a recurrent event regardless to the site of placental implantation in the subsequent pregnancy. Collectively, these findings support the notion that a preterm delivery in women placenta previa has the epidemiologic characteristic of spontaneous preterm birth. Thus, patients with placenta previa who had an early preterm delivery may need to be treated as patients with a previous spontaneous preterm birth in term of perinatal counseling and preventive measures in their subsequent pregnancies. The approach regarding women with placenta previa who delivered at late preterm is not that clear since at these gestational age some of the deliveries may be indicated and dependent on physician decision.

## Competing interests

On behalf of me and all of the co-authors I declare that we have no known conflict of interest regarding this manuscript what so ever.

## Authors' contributions

OE designed the study and performed the statistical analysis and drafted the manuscript; LN performed the statistical analysis and contributed to the manuscript; VK, IEW, and RBW participated in writing the manuscript; DD and MM participated the design of the study and helped to draft the manuscript. All authors read and approved the final manuscript.

## Pre-publication history

The pre-publication history for this paper can be accessed here:

http://www.biomedcentral.com/1471-2393/12/82/prepub
